# The Spectrum of Psychiatric Pathology in a Patient with Genetically Verified Huntington's Disease

**DOI:** 10.1155/2015/742471

**Published:** 2015-09-16

**Authors:** Samir Alkabie, Daljinder Singh, Amy Hernandez, Rhaisa Dumenigo

**Affiliations:** ^1^Department of Psychiatry, Larkin Community Hospital, Miami, FL 33143, USA; ^2^Saba University School of Medicine, Devens, MA 01434, USA

## Abstract

Psychiatric and behavioral disturbances are common in Huntington's disease (HD) and contribute significantly to its morbidity and mortality. We herein present the case of a 43-year-old woman with genetically verified HD, whose deteriorating psychiatric condition necessitated multiple inpatient psychiatric hospitalizations and featured a clinical spectrum of neuropsychiatric disturbances classically associated with HD. This paper reviews the literature concerning Huntington's psychopathology and provides an illustrative case example of its clinical nature.

## 1. Introduction

HD, historically termed Huntington's chorea [1], is an autosomal dominant neurodegenerative condition, known for producing a hyperkinetic, choreiform (dance-like) movement disorder, as well as cognitive decline and neuropsychiatric disturbances [2, 3]. Its genetic etiology is related to an unstable and expanded CAG trinucleotide repeat within the HD gene (also referred to as* Huntingtin*,* HFF*, or* IT15*) on chromosome 4p16.3, which encodes a mutant Huntingtin (mHtt) protein with polyglutamine expansion [4]. Neuropathologically, HD is characterized by progressive and selective neuronal loss in the basal ganglia (i.e., caudate and putamen), centromedian thalamic nuclei, and cerebral cortex associated with atrophy, inflammation, and gliosis, which disrupts the basal ganglia thalamocortical circuit [5–9]. The disconnection between orbitofrontal, anterior cingulate, and lateral prefrontal cortices and striatum (frontal-subcortical circuitry) is particularly pronounced and thought to underlie many of the cognitive, affective, and behavioural disturbances in HD [10, 11]. Striatal medium spiny neurons, containing *γ*-aminobutyric acid (GABA) and enkephalin, are the primary cellular target of HD, affected early in its disease course [12, 13]. The exact mechanisms by which mHtt induces steady neurodegeneration in HD remain incompletely understood, but may relate to misfolding, aggregation, and reduced clearance of the mutant protein causing progressive cerebral degeneration starting in the caudate nucleus and putamen [14]. On the one hand, intranuclear and cytoplasmic aggregations of mHtt in striatal neurons are thought to be neurotoxic [15]; and on the other hand, mHtt inclusion bodies predict improved neuronal survival by sequestering toxic mHtt away from the rest of the neuron [16]. Further research is required to better discern whether mHtt aggregation is helpful or harmful in the molecular pathogenesis of HD.

The clinical onset of HD—usually defined by age of motor onset—typically occurs between the fourth and fifth decades of life and inversely correlates with number of CAG repeats [17, 18]. Early motor symptoms such as twitching, clumsiness, discoordination, and inevitable progress to facial contortion and grimacing; jerky, arrhythmic, and asymmetric gait; twisting and writhing whole body movements; and dysphagia, rigidity, dystonia, and hypokinesia [12]. Patients often succumb to their illness 15–20 years after motor onset [19], most commonly due to aspiration pneumonia or cardiovascular disease [20, 21]; however, 25% of HD patients attempt suicide and 8-9% succeed at it, representing a significant cause of death among HD patients [22]. Although, onset is clinically defined by motor presentation, subclinical affective symptoms manifest approximately 10 years before motor signs in a third of HD gene carriers [23]. In many patients, the earliest marker of HD is neurobehavioral [23].

The neuropsychiatric burden of HD has been well recognized since 1872 in George Huntington's original essay “*On Chorea*” [24], wherein he describes the propensity of choreic patients toward insanity and suicide. Studies have since validated these early observations, reporting, among HD patients, high rates of apathy, depression, anxiety, and irritability [2], as well as suicide rates eight times that of the general population [25]. In a large, European prospective HD cohort (REGISTRY), 73% of participants exhibited some form of psychopathology, the most prevalent of which were depression, irritability/aggression, and obsessive compulsive behaviors (OCBs) at all stages of HD and apathy at advanced stages [19]. Psychosis remains a relatively rare clinical phenomenon in HD, usually expressed after the full clinical syndrome is apparent, and more often as delusions rather than hallucinations [2, 26, 27]. We herein present the case of a woman with verified HD, who expressed a spectrum of neuropsychiatric disturbances classically associated with HD.

## 2. Case Report

A 43-year-old woman presented to the emergency department with acute agitation after she became violent and struck her mother, breaking objects throughout the house. The patient had a previous positive genetic test result for HD and a history of multiple inpatient psychiatric admissions and had been discharged from a regional psychiatric unit four days prior for impulsive outbursts of anger and aggression, along with numerous suicide attempts. The patient's home medications were chlorpromazine 50 mg daily, clonazepam 1 mg every 8 hours, divalproex 500 mg three times a day, and gabapentin 300 mg every 8 hours. Haloperidol 5 mg twice a day in the emergency department did not adequately control her impulsivity and agitation. She was involuntarily admitted to the inpatient psychiatric unit for close observation and medical management. The patient was discharged after ten days to her private home and returned two days later escorted by police, after attempting suicide by ingesting shampoo, hairspray, and toothpaste and expressing suicidal ideations. On admission, she appeared unkempt and withdrawn, resistant and uncooperative, whispering to herself, exhibiting bizarre behavior such as ritualistic muttering of statements under her breath, alogia, avolition, loss of social interest towards her surroundings, and an unwillingness to engage in eye contact or conversation.

According to her mother and maternal half-sister, the patient was perfectly normal five years ago, formerly trained and employed as a paralegal with four children. However, after the birth of her youngest child, a now five-year-old girl, the patient began to manifest symptoms of severe depression which progressively worsened to where she would cry inconsolably. The patient sometimes became verbally and physically abusive towards her family. The first motor symptoms appeared a year later with hand tremors and grasping difficulties, followed by gait imbalance. Eventually, the patient required assistance to manage her activities of daily living and had to move in with her mother, thereafter demonstrating progressively worsening motor symptoms, depression, mood lability, irritability, agitation, anxiety, and episodically violent behavior. The patient's mother had difficulty trying to control her behavior and had to call the police on several occasions so her daughter could get medically evaluated.

The patient's mother reported no family history on her side of abnormal movements or impulsive behavior. The patient's biological father emigrated to the United States (US) from Cuba in his early 50s. The mother recalled that the father at that time, about twenty years prior, had similar episodes of depression, anger outbursts, and abnormal movements. She observed him to be very nervous, anxious, and restless, constantly getting up from a seated position and had to be hospitalized several times at an inpatient psychiatric unit to stabilize his mood and behavior. The patient has two paternal half-siblings in Cuba who could not be contacted for their medical history and two maternal half-siblings in the US who are symptom-free. Although not confirmed, it appears this patient inherited the disorder from her father, who may have been the index case of HD in their family pedigree, based on the lack of symptomatic family history prior to her biological father.

**Figure 1:**
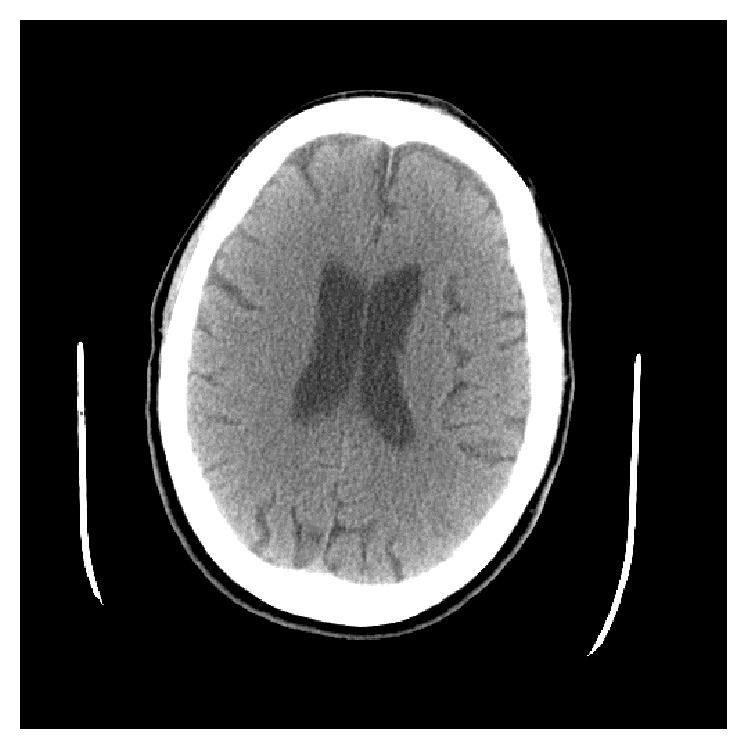
Axial brain computerized tomography exhibiting diffuse cortical atrophy, prominent sulci, and ex vacuo ventricular enlargement.

While admitted in the inpatient psychiatric unit, the patient characteristically paced with a subtle choreiform gait, exhibiting unpredictable mood lability, confrontational behavior, and sporadic tearfulness. She sometimes became verbally abusive towards staff, yelling profanity. The patient communicated, on several occasions, the delusion that her husband had just visited her at the hospital, when he had not. She also fixated on the false belief that her husband and family were taking her home, despite no visits or calls from her husband or family. In addition, although not formally assessed, the patient had memory lapses. She would forget eating soon after she ate. She had obsessive thinking about “cleanliness” and a compulsive need to repeatedly shower, observed wandering the psychiatric ward with almost persistently damp hair, a behavior that resulted in a fall and head injury in the shower requiring neurological workup. The head computerized tomography from this workup revealed no fractures, only orbital soft tissue swelling, as well as imaging features compatible with HD neurodegeneration, including diffuse cortical atrophy, prominent sulci, and ex vacuo dilation of the ventricles (Figure 1). The diagnosis of HD was verified by a genetic assay that subjected whole blood DNA to polymerase chain reaction (PCR) amplification and electrophoresis size analysis, quantifying the number of CAG repeats in the HD gene with sensitivity and specificity of 99% (ARUP Laboratories Inc., Salt Lake City, UT). The patient had one HD allele in the normal range at 17 CAG repeats and the other allele in the affected range at 45 CAG repeats, verifying the diagnosis of HD.

The patient was admitted and discharged a total of eleven times in five months, treated for numerous exacerbations of depression, anxiety, irritability, aggression, and suicidal and psychotic behavior, requiring acute inpatient stabilization. She was treated with atypical antipsychotics (quetiapine and olanzapine), typical antipsychotic (haloperidol), antidepressants (venlafaxine and escitalopram), mood stabilizers (lithium and divalproex), and benzodiazepines (clonazepam and lorazepam). The patient was encouraged to participate in group therapy and psychiatric treatment aimed at mood stabilization and building coping skills to better cope with chronic terminal illness. The patient responded well to a regimen of olanzapine 15 mg at night, escitalopram 20 mg daily, lithium carbonate 300 mg in the morning and 600 mg at night, divalproex sodium 500 mg twice a day, and clonazepam 1 mg twice a day. She reached the maximum benefit of treatment in the inpatient psychiatric unit while being compliant with medications and psychiatric therapy, as evidenced by (1) diminished impulsivity and aggressive behavior; (2) improved mood and affect; (3) improved interpersonal interactions with peers and nursing staff; and (4) reassuring reports that she was eating and sleeping well without any acute physical or psychiatric complaints. The patient was monitored closely for adverse effects of the medications with no reported emergent adverse effects and was eventually transferred to a state mental health long-term care facility.

## 3. Discussion

We report on a woman with genetically verified HD, whose psychiatric health precipitously deteriorated in five years, resulting in the severe expression of depression, anxiety, suicidality, irritability/aggression, obsessive compulsive symptoms, cognitive decline, and psychosis. These psychiatric manifestations along with motor and cognitive disturbances became more prevalent over time and devastated her quality of life, resulting in unemployment, separation from family, and multiple psychiatric inpatient hospitalizations. The behavioral problems of this patient arguably had a greater impact on her quality of life than her motor and cognitive dysfunction, emphasizing the importance of research aimed at limiting the damage caused by HD psychopathology.

Depression is one of the most common psychiatric features of HD and manifests early in its disease course [23, 28]. Its prevalence ranges between 33 and 69% [2] in HD compared with 15% in the general population [29]. Although depression often predates motor symptoms [30, 31], the prevalence of depression typically peaks one year after clinical onset of motor symptoms [28]. This timeframe corresponds well with the relative manifestations of affective and motor symptoms in our case patient, whereby depressive symptoms predated motor symptoms and intensified after motor onset.

Several studies have reported relatively high rate of depression among HD patients. In a retrospective study, Paulsen et al. found that rates of depression were twice that of the general population [32]. Similarly, a cross-sectional study demonstrated that rates of major depressive disorder in presymptomatic (18.2%) and symptomatic (16.5%) HD patients were twice that of noncarrier controls (7.1%, *P* < 0.001) [33]. Likewise, a longitudinal prospective study (TRACK-HD) reported a significantly higher incidence of depression in individuals with HD compared with their spousal controls [34]. There are studies that suggest depression diminishes with HD progression. In a retrospective study, Paulsen et al. grouped 2835 HD patients by their disease stage and found that the prevalence of depression was highest at stage 2 HD, declining at later stages [32]. The authors contended that cognitive impairment at advanced HD stages most likely diminishes subjective awareness of depression and thus explains the declining incidence of depression at later disease stages [32].

The underlying mechanisms of affective disorder in HD could have a neurobiological etiology, in which neostriatal damage acts as a nidus for behavioral aberrations [35]; however, the psychological reaction to being at risk for HD, growing up in an insecure environment, and becoming aware of disease onset could equally contribute to the psychopathogenesis of affective disorder [2]. Familial clustering of depression may exist, such that the genetic background of the individual with HD may predispose them to developing depression during the course of their disease [28]. Folstein et al. identified genetic heterogeneity in HD relative to its association with depression. Their study demonstrated that the association between MDD and HD was confined to certain families with a strong history of affective disorders [31]. The HD gene carriers within these families had a significantly higher rate of depression than noncarriers [31]. The patient in our case report suffered from severe major depression at onset in her 30s which persisted throughout the course of her illness. Her biological father suffered a similar presentation of affective disturbances in his 50s, consistent with the notion that heritable factors influence the relative risk of depression in HD gene carriers. Thus, family history of affective disorders could be an important risk factor for developing depression in early stages of HD.

Anxiety was prevalent in our patient and refractory to treatment with antidepressants and benzodiazepines. However, anxiety in HD has not been a major focus of research [2]. Studies of anxiety in HD are embedded within research of depression in HD, with anxiety, as a factor, often combined with depression. A factor analysis of Unified Huntington's Disease Rating Scale (UHDRS) behavioral data from the European Huntington's Disease Network REGISTRY study combined anxiety with depression and confirmed their correlation with HD [36]. Olanzapine 5 mg improved symptoms of anxiety, as well as depression, irritability, and obsessive and compulsive behavior in a small open-label clinical study treating eleven patients with HD [37]. Recently, a systematic review of anxiety in HD reported that the relative prevalence of anxiety in manifest HD ranges between 13 and 71% [38]. The rates of anxiety were no different in premanifest versus manifest HD gene carriers and had no correlation with measures of disease progression [38]. Anxiety was, however, associated with psychometric measures of depression, suicidality, irritability, quality of life, pain, illness beliefs, and coping styles [38]. Future studies are needed to elucidate the determinants of and therapeutic strategies for anxiety in HD. Randomized controlled trials are required to evaluate effective and safe therapeutic strategies for targeting depression and anxiety in HD patients.

Suicidality plays a major role in the morbidity and mortality associated with HD, with a quarter of HD patients attempting suicide and rates of suicide between five and ten percent [28]. Paulsen et al. studied 4,171 HD patients and found that stage 2 HD patients had both the highest incidence of depression and the highest rate of suicidal ideations (21.6% in stage 2 versus 16.7 in stage 1). The risk doubled from 9.1% in at risk patients with normal neurological exam to 19.8% in patients with soft neurological signs, peaking to 23.5% in patients with “possible HD.” Paradoxically receiving the diagnosis of HD does not worsen and may reduce the risk of suicide. The risk declined to 16.7% once a definitive diagnosis was attained, emphasizing the important role of timely genetic testing in reducing risk of suicide in patients with a positive family history, at risk for HD [39]. Therefore, it is critical to assess suicide risk immediately prior to genetic testing and when independence is threatened [39]. The patient in our case report suffered the brunt of her psychiatric instability and suicide attempts living in her mother's care, dependent and unable to palliate her own activities of daily living. It was under these circumstances, when her independence was threatened, that her psychiatric stability suffered most, leading to severe depression and multiple suicide attempts and outbursts of behavioral instability.

Irritability/aggression without prior history of short temper is a hallmark of HD psychopathology, causing considerable distress to patients and their relatives [2, 40]. In a large prospective cohort (REGISTRY), the prevalence of irritability and aggression increased with disease stage (10.4% in stage 1 versus 19.6% in stages 4-5) [19]. The patient in our case report developed a* de novo* propensity for outburst of aggression on a background of irritability, which peaked around five years after the onset of her affective and neurological symptoms. These outbursts were at times so severe they led to violence, damaged personal relationships, and police escorts to the hospital for involuntary inpatient psychiatric confinement. This expression of inappropriate behavior is thought to result from impaired integration of limbic and emotional input into behavioral responses, secondary to orbitofrontal-striatal neurocircuitry defects, which initially manifest as irritability and later as aggression [2, 41].

OCBs are an important psychiatric disturbance encountered in HD patients, whereby obsessive thoughts and feelings compel the affected individual to perform repetitive, purposeful rituals to balance their obsessions. In the REGISTRY cohort, 25.8% of HD patients had OCBs with 13.2% exhibiting moderate to severe symptoms [19]. The patient in our case report had obsessive and compulsive psychiatric features, namely, contamination obsessions and compulsive showering. She showered so frequently it posed a significant fall risk, resulting in one such event. She also isolated herself from others, likely to balance her compulsion towards behaving aggressively. However, with psychiatric stabilization by pharmacotherapy and psychotherapy, the patient was gradually able to participate more in group therapy and interact with staff and peers.

OCBs more often affect HD patients later in their disease course. Beglinger et al. [42] studied the relationship between obsessive compulsive symptoms and HD in a large HD gene-carrier cohort (*n* = 3964) and found that the prevalence of obsessive compulsive symptoms in patients with advanced disease with functional disability was threefold greater than at risk patients without motor symptoms (24 versus 7% obsessions and 12 versus 3.5% compulsions for stage 4 versus stage 0, resp.) [42]. It is thought that obsessive compulsive disorder (OCD) and HD both involve frontal-striatal dysfunction resulting from similar neuropathology involving the basal ganglia and frontal-striatal circuits. Huntington's chorea is characterized by frontal-striatal disinhibition due to loss of inhibition from the internal globus pallidus to the thalamus. This mechanism, along with decreased dorsolateral prefrontal activity, characteristic of OCD, underlies the disinhibited repetitive behavior common to both HD and OCD [43].

Cognitive decline in HD mainly pertains to impaired executive functioning, such as working memory and response inhibition [43]. Studies using functional positron emission tomography imaging from the mid-1980s revealed that brains of HD patients exhibit frontal and striatal hypometabolism and thalamic hypermetabolism [44]. Thus, cognitive domains that depend on frontal-striatal function such as working memory are hypofunctioning in HD patients. The patient in our case report frequently demonstrated poor working memory, often forgetting whether she had eaten, remarking she had not eaten when she had in fact eaten shortly beforehand. The early cognitive decline observed in HD is thought to be driven by changes in cortical cytoarchitecture secondary to altered corticosubcortical connectivity secondary to primary basal ganglia degeneration, excessive thalamocortical facilitation, and frontal-striatal dysfunction [11, 45].

Psychotic symptoms in HD more frequently manifest as delusions, characterized by fixed, false, and irrational beliefs with no basis in reality, rather than hallucinations, that is, sensory perceptions that are not real [46]. In a multicentered, prospective clinical study of 960 patients with definite HD, 5.4% had delusions and 1.3% had visual or auditory hallucination [27]. Paulsen et al. reported 11.5% delusions versus 1.9% hallucinations in 52 HD patients who were subjected to the neuropsychiatric inventory [26]. In the REGISTRY cohort, 4.1% of HD mutation carriers had psychotic symptoms [19]. The patient we reported on had a diagnosis of psychosis due to HD with delusions. Her delusional psychosis emerged sporadically, predominantly centered around the false belief that her husband and family had visited her in the hospital and were taking her home. Overall, psychosis in HD is much less common than depression, anxiety, and aggression but has a devastating impact on daily functioning and quality of life [40].

Studies have shown that early-onset HD confers higher risk of eventually developing psychotic features compared with later onset disease [47–49]. Some studies show that families who carry the HD gene also have a higher incidence of psychosis. A study of 44 HD patients demonstrated that those with psychosis were more likely to have a first-degree relative with psychosis, eluding to the heritability of psychosis in HD [50, 51]. Thus, a family history of HD and psychosis may be an important risk factor for developing HD psychosis [50, 51]. We were unable to determine whether the biological father of the patient in our case report had psychotic features during his illness, but such a familial trend is likely given what is known about familial clustering of HD psychosis.

## 4. Conclusion

The psychiatric manifestations of HD contribute a great deal to the difficulties these patients face during the course of their illness. Their symptoms encompass a spectrum of psychiatric disorders, including depression, anxiety, suicidality, irritability/aggression, obsessive compulsive behaviors, and psychosis, each of which may require targeted symptomatic neuropsychopharmacological management.
